# Immunohistochemical localization of *Cassava brown streak virus* and its morphological effect on cassava leaves^☆^

**DOI:** 10.1016/j.pmpp.2018.06.001

**Published:** 2019-01

**Authors:** Maliha H. Saggaf, Joseph Ndunguru, Fred Tairo, Peter Sseruwagi, José Trino Ascencio-Ibáñez, Dora Kilalo, Douglas W. Miano

**Affiliations:** a Mikocheni Agricultural Research Institute, P.O. Box, 6226, Dar-es-Salaam, Tanzania; b University of Nairobi, P.O. Box 30197-00100, Nairobi, Kenya; c North Carolina State University, Raleigh, NC, 27695, USA

**Keywords:** Manihot esculenta, Cassava brown streak virus (CBSV), Immunohistochemistry, Cassava brown streak disease, Immunolocalization

## Abstract

The localization of *Cassava brown streak virus* (CBSV) in cassava (*Manihot esculenta*) leaf tissues was determined and cellular morphological changes in CBSV-infected tissues were evaluated. CBSV-symptomatic leaves were screened with CBSV-specific primers using reverse-transcriptase polymerase chain reaction. Immunohistochemical reactions showed precipitation in CBSV-infected but not CBSV-free tissues, demonstrating successful localization of CBSV. Microscopic inspection showed significantly larger (*P* < 0.001) midribs in CBSV-infected compared with control (uninfected) leaves. Viral accumulation occurred in middle and lower but rarely in young upper leaves. This immunohistochemical method for virus localization will be invaluable for efficient screening of CBSV and for breeding resistant cassava.

## Highlights

• Immunohistochemistry can be used to localize CBSV within infected leaf tissues.
• CBSV is frequent in abaxial and adaxial epidermal cells but rare in the phloem.
• CBSV infection influences the cellular morphology of the infected leaf tissues.

## Abbreviations

ACMV: African cassava mosaic virus
ANOVA: Analysis of variance
BHUA: Biotinylated horse universal antibody
CBSD: Cassava brown streak disease
CBSV: Cassava brown streak virus
DFID: Department for International Development
EACMV: East African cassava mosaic virus
PBS: Phosphate buffered saline
PCR: Polymerase chain reaction
SEM: Standard errors of the means
UCBSV: Ugandan cassava brown streak virus
WAP: Weeks after planting
ABC: Avidin–biotin complex detection system
AEC: 3-amino-9-ethyl carbazole detection system
CTAB: Cetyltrimethylammonium bromide
DAPI: 4′–6′diamidino-2-phenylindole
IHC: Immunohistochemistry
RT: Reverse transcriptase

## 1 Introduction

Cassava (*Manihot esculenta* Crantz, Euphorbiaceae) can be grown year-round in Africa and provides a consistent food source in periods when other crops cannot be grown, making it an important food staple for over 800 million people across the tropics and sub-tropics [1]. The crop is cultivated easily, has low input requirements, tolerates low rainfall and poor soils, and is propagated easily through stem cuttings; thus, it is a particularly valuable crop among the poor [2]. Two viral diseases, cassava brown streak disease (CBSD) and cassava mosaic disease, threaten cassava productivity in Africa, causing major yield losses (up to 100%) in susceptible varieties. These diseases drastically affect food security on the African continent [3].

CBSD, caused by *Cassava brown streak virus* (CBSV) and *Ugandan cassava brown streak virus* (UCBSV), was previously known to be endemic in low-altitude areas of coastal East Africa where it has low incidence and severity [3]. In contrast, where it infects plants across sub-Saharan Africa, CBSD limits cassava productivity [4] and is emerging as the most serious challenge to cassava cultivation [5]. The threat posed by CBSD appears to be growing; regional surveys of cassava disease and other pests have confirmed the rapid spread of CBSD across East and Central Africa [3,6,7].

Maruthi *et al*. [8] reported that whiteflies, *Bemisia tabaci*, transmit CBSD (both CBSV and UCBSV) at a maximum rate of 22% under controlled conditions. *B. tabaci* is currently the only experimentally proven vector for the disease. Similar to other ipomoviruses, CBSV and UCBSV are transmitted semi-persistently and the virions are not retained for more than 24 h [9]. Successful and efficient mechanical transmission of CBSV and UCBSV by sap inoculation to the test plant *Nicotiana benthamiana* was shown by Ogwok *et al*. [10] and therefore sap inoculation was determined to be suitable for infectivity assay experiments. However, mechanical transmission of the two viruses is completely ineffective on cassava itself, and to date, infectious clones of the two pathogens are not available. So far, the only reliable transmission methods of CBSV and UCBSV between infected and non-infected cassava plants are grafting between cassava plants [11] and leaf harvesting. Mohammed *et al*. [12] and Wagaba *et al*. [13] demonstrated higher virulence of CBSV than UCBSV in grafting experiments; CBSV-infected cuttings showed significant reduction of sprouting resulting from elevated virus accumulation compared with UCBSV infected cuttings.

CBSD foliar symptoms are characterized by feathery leaf chlorosis that first appears along the margins of veins and later develops into chlorotic blotches [10,14]. CBSV is a positive-sense single-stranded RNA virus belonging to the family *Potyviridae* and the genus *Ipomovirus* [15]. The virus encodes a large polyprotein cleaved into 10 mature proteins [16]. A study conducted by Mbanzibwa *et al*. [17] revealed the complete genome of CBSV consists of 9069 nucleotides (nt) excluding the poly (A) tail that encodes 9 of the 10 expected proteins of the family *Potyviridae.* Studies have shown that foliar symptoms caused by CBSD and their associated cellular modifications depend on whether the plant is infected by CBSV alone or a double infection of both CBSV and UCBSV, which may result in synergistic interactions [10], although evidence of synergistic interactions has not been verified by field surveys.

Immunohistochemistry (IHC; also known as immunolocalization) is a molecular detection technique based on the ability of antibody molecules, especially immunoglobulin G, to recognize and bind to an antigen against which they were raised in an experimental animal [18]. IHC was first described in 1942 by Coons *et al*. [19] when they reported the localization of pneumococcal antigen in liver sections using a fluorescent-labeled antibody. IHC has been extensively used and refined to study and locate proteins in animal cells and tissues [20], but is seldom used on plants [21]. IHC was first applied to plants in 1970 with the location of antigens of the cell wall in pollen grains [22] and the proteins in cotyledons [23]. IHC has also been used to detect *Tomato golden mosaic virus* in infected *Nicotiana benthamiana* leaves [24] and to detect the viral replication protein AL1 in *Cabbage leaf curl virus*-infected leaves of *Arabidopsis thaliana* [25].

IHC makes use of epitopes found on antigens, which antibodies are specific to. Here, we used primary antibodies specific to the CBSV coat protein (rabbit-*anti*-CBSV CP), that were provided by the Mikocheni Agricultural Research Institute (MARI) in Tanzania. The antibodies were produced from rabbit and used with a secondary antibody, biotinylated horse universal antibody (BHUA), to detect CBSV in infected cassava leaves. We used the avidin–biotin complex detection system (ABC) [26], in which the secondary antibody is labeled with an enzyme that, upon incubation with a substrate, causes precipitation of insoluble colored products. This process forms a binding complex in which rabbit-*anti*-CBSV CP binds to CBSV epitopes, and is then recognized by the BHUA. Selection of a substrate for precipitation depends on the enzyme labeled on the secondary antibody; in this case, the enzyme horseradish peroxidase was used. The 3-amino-9-ethyl carbazole (AEC) system was used as the substrate, and reacted with the enzyme to form a precipitate.

Plant viruses are typically identified and characterized using molecular studies based on their genomic structure, or by their morphology using electron microscopy [27]. Plant virus detection, identification, and localization methods that are specific, sensitive, and relatively inexpensive, have been developed for plant research [28]. Virus–plant host interaction studies show that plant viruses can be important biotechnological tools in plant species [27], causing devastating diseases [3]; however, the cellular effects of CBSV-infected cassava plants have not been fully explored. For example, there is insufficient information to explain the linkage between CBSD development and CBSV localization within the tissues of infected plants.

Our study aims to explore the localization of CBSV in cassava tissues and examine the cellular and morphological changes induced by the virus using immunohistochemical methods. One challenge to understanding virus location in plant tissues is the ability of the putative vector to move within the plant and be transmitted to other plants. The ability to locate the virus in plant tissues is critical to understanding how a virus moves and where it ends may help with control practices in the future. A clear understanding of the distribution of a virus in an infected plant can also help direct diagnostic sampling, which would facilitate accurate and timely diagnostics.

Here, we predict that localizing CBSV using the coat protein of the virus will reveal the distribution of viral particles in the cells and tissues of infected cassava. Information on the localization of CBSV will be invaluable to investigating viral transport mechanisms and understanding the complexity of virus–plant interaction as well as designing virus diagnostic tools and protocols. Studying pathogen movement among plant cells is an important and essential step that will complement other approaches to studying virus pathogenicity. The information generated here will support future efforts for CBSD control.

## 2 Materials and methods

### 2.1 Sample collection and establishment

A total of 14 whole leaf samples from CBSD-symptomatic susceptible cassava variety Kibandameno and their corresponding cuttings were collected - sterilizing the blade after each use - from the Sugarcane Research Institute-Kibaha and Chambezi substation in Bagamoyo, both in the Coastal Region of Tanzania. Collected leaves were pressed in brown envelopes and transferred to MARI for further analysis. Cuttings were established in 5-L pots in an insect-proof screen house (2-μm mesh, temperature range of 28–35 °C, and 10-h daylight exposure) at MARI for regeneration. Samples lacking CBSD-like symptoms were also collected and used as negative controls.

### 2.2 Screening for CBSV and cassava mosaic begomoviruses

#### 2.2.1 Nucleic acids extraction

The buffer for extraction of nucleic acids comprised 2% (w/v) cetyltrimethylammonium bromide (CTAB), 100 mM Tris-HCl, 20 mM EDTA, 1.4 M NaCl, and 715 mM β-mercaptoethanol; reagents were mixed just before use. Total nucleic acids were extracted from leaf samples using a modified CTAB method described by Chang *et al*. [29], with the following modification: 60 mg of leaf samples were ground in 1 mL of CTAB extraction buffer in mortars instead of in liquid nitrogen. Nucleic acid quality and integrity were verified by gel electrophoresis using 1.5% agarose gel stained with 0.1 mg/mL of ethidium bromide in 100 mL of 1× Tris-acetate-EDTA (TAE) buffer solution. The gel was viewed under ultraviolet (UV) light using a BioDoc-It^®^ 210 Imaging System (Thomas Scientific, Swedesboro, NJ, USA).

#### 2.2.2 cDNA synthesis

The complementary strand of DNA (cDNA) was synthesized using oligo (dT)_25_ (ThermoFisher Scientific, Waltham, MA, USA). Total RNA extracted was normalized to 200 ng/μl and mixed with 6.4 pmol/μl oligo (dT)_25_ and 1 mM dNTPs (ThermoFisher Scientific), then incubated at 65 °C for 5 min. Then, 0.5× RT buffer (ThermoFisher Scientific), 1 U/μl RNase inhibitor (ThermoFisher Scientific), and 10 U/μl RT NxGen™ M-MuLV (Lucigen, Middleton, WI, USA) were added to the mixture and topped up with nuclease-free water to a total volume of 20 μl. Polymerase chain reaction (PCR) conditions were set at 42 °C for 50 min and then at 85 °C for 5 min using a GeneAmp^®^ PCR System 9700 thermocycler (ThermoFisher Scientific).

#### 2.2.3 PCR amplification of CBSV and cassava mosaic begomoviruses

Synthesized cDNA was screened for the presence of CBSV and UCBSV by conventional PCR using primers developed by Mbanzibwa *et al*. [15] (Table 1). PCR reactions consisted of 200 ng of cDNA as template, 0.1 mM dNTPs, 0.4 μM of forward and reverse primers (CBSDDF2 and CBSDDR) amplifying both CBSV and UCBSV, 1.25× PCR reaction buffer + 2 mM MgCl_2_ (ThermoFisher Scientific, Waltham, MA, USA), and 1 U *Taq* DNA polymerase (ThermoFisher Scientific) topped up to 25 μl with nuclease-free water. PCR for amplifying CBSV and UCBSV was run on the thermocycler using conditions as follows: denaturation of 95 °C for 10 min, 35 cycles consisting of 95 °C for 15 s, 51 °C for 30 s, 72 °C for 30 s, and a final extension stage of 72 °C for 10 min.

**Table 1:** Primers used in the study to target four types of cassava-infecting viruses.

Primer	Sequence (5′–3′)	Target^a^	Size (bp)	Reference
CBSDDF2	GCTMGAAATGCYGGRTAYACAA	CBSV UCBSV	344	[15]
CBSDDR	GGATATGGAGAAAGRKCTCC		440	
JSP001	ATGTCGAAGCGACCAGGAGAT	ACMV	774	[30]
JSP002	TGTTTATTAATTGCCAATACT			
EAB555F	TACATCGGCCTTTGAGTCGCATGG	EACMV	556	[30]
EAB555R	CTTATTAACGCCTATATAAACACC			

a CBSV, *Cassava brown streak virus;* UCBSV, *Ugandan cassava brown streak virus;* ACMV, *African cassava mosaic virus;* EACMV, *Eastern African cassava mosaic virus*.

For quantification purposes, the PCR products were electrophoresed on 1% agarose gel and viewed under UV light using the gel documentation machine (BioDoc-It^®^ 210 Imaging System). The results of the screening were used to tag the established cassava cuttings.

*East African cassava mosaic virus* (EACMV) and *African cassava mosaic virus* (ACMV) were screened using the primer pairs EAB555  F/R and JSP001/002, respectively [30]. The PCR master mixes were of the same concentrations and consisted of 1× PCR reaction buffer +2 mM MgCl_2_, 0.1 mM dNTPs, 0.04 U *Taq* DNA polymerase, 0.4 μM of both forward and reverse primers, and 200 ng of DNA of samples prepared topped up to a total volume of 25 μl with nuclease-free water. Amplification used the following parameters: an initial denaturation stage of 94 °C for 3 min, followed by 30 cycles of 94 °C for 45 s, 56 °C for 45 s, and 72 °C for 1 min, and a final extension step of 72 °C for 7 min, using the thermocycler. The PCR products were run for 45 min at 120 V on 1% agarose gel and viewed under UV light as previously described to verify the presence or absence of the viruses.

CBSV-infected and healthy cassava cuttings were divided into pieces and planted in separate screen rooms into 15 pots in the screen house at MARI. Cuttings infected with UCBSV, ACMV, EACMV, or co-infected with CBSV and UCBSV were discarded from the study. To minimize the potential for virus competition during amplification, we took the following steps: (i) we normalized the starting concentrations of materials; (ii) we terminated PCR at 30 cycles and 35 cycles to minimize non-specific amplification, as has been discussed in McCullock *et al*. [31]; and (iii) we used primer pairs (CBSDDF2/CBSDDR; Mbanzibwa *et al*. [15], EAB555  F/R and JSP001/002 [30]) that have been used extensively for CBSV and UCBSV diagnostics and have confirmed efficiencies for simultaneous detection of both viruses. Given these protocols, we are confident the chances for viral competition was low.

### 2.3 Leaf sample preparation, embedding, and sectioning

Leaf samples from healthy leaves and leaves symptomatic of infection with CBSV were collected from the established tagged plants in the screen house. Leaf samples were collected 4 weeks after planting (WAP) for virus-localization studies. To study morphological differences, sampling was done a second time, 6 WAP, and targeted three leaf positions: newly opened, middle, and mature lower leaves, which are referred to here as top, middle, and bottom leaves, respectively. The samples were held flat in envelopes to maintain leaf integrity and immediately transferred to the laboratory. The integrity of the samples was further maintained by fixing them as previously reported [32] using 4% formaldehyde in 1× phosphate buffered saline (PBS) and incubating them at 4 °C overnight in 5-mL glass vials (Wheaton, DWK Life Sciences, Millville, NJ, USA). The next day, the samples were washed twice with 1× PBS. Samples were then embedded in 5% agarose dissolved in 1× PBS. Using a VT1000 S vibratome (Leica Biosystems, Buffalo Grove, IL, USA), we made 100-μm cross-sections.

### 2.4 IHC detection of CBSV from infected cassava leaf tissues

Immunohistochemical staining was performed on cassava cross-sections following the protocol described by Shen and Hanley-Bowdoin [32], with minor modifications. Chlorophyll was removed from cross-sections using 100% methanol. The leaves were then quenched using peroxidase quenching solution (3% hydrogen peroxidase in 100% methanol) for 30 min on a nutator (Stuart Gyro-Rocker, Sigma-Aldrich, Hoxter, Germany) to prevent false positive and high background levels. 1× PBS-2% BSA (PBS-BSA) was used to wash away the quenching solution five times for 5 min each. Non-specific sites in the tissues of samples were blocked using a blocking solution containing 1.5% universal horse serum in 1× PBS, 0.1% Tween 20, and 2% BSA (PBS-T-BSA) and incubated at room temperature for 1 h on a nutator to prevent further background staining. Using PBS-T-BSA, the samples were washed carefully three times, and then purified with the primary antibody solution obtained from MARI (rabbit-*anti*-CBSV CP) which was applied and incubated for 1 h at room temperature on a nutator. For optimization, the antibody was made at several dilutions – 1:1000, 1:2000, 1:3000, 1:5000, 1:7500 and 1:10000 PBS-T-BSA. Samples were washed again with PBS-T-BSA to remove unbound antibodies and antibodies weakly bound to non-specific targets.

Next, a secondary antibody— BHUA (5:15:1000 BHUA:serum:PBS-T-BSA)—was added and allowed to incubate at room temperature for another 1 h on a nutator. The samples were then washed with PBS-T-BSA, and then with 1× PBS on the nutator for 5 min each. The ABC solution (Vector Laboratories, Burlingame, CA, USA) was prepared and allowed to stand for 30 min at room temperature, then added to the samples. Samples were placed on a nutator for 1 h, then washed three times with PBS for 10 min on the nutator, and then the AEC solution was added. Color development was optimal after 3 min, after which samples were washed with 100% ethanol for 3 s and with PBS for 5 min. Finally, samples were stored in sectioning baskets containing sterile double-distilled water at 4 °C.

### 2.5 Sample mounting and microscopy examination

A 4′-6′-diamidino-2-phenylindole (DAPI) solution [24] was prepared by mixing 0.001 mg/μl DAPI, 1× PBS, and 90% glycerol in 1× PBS in a total volume of 100 μl. Approximately 100 μl of DAPI solution was dispensed at the center of a clean microscope slide, Next, using a toothpick, sections of leaf tissue were placed carefully in the DAPI solution. The toothpick was used to spread the leaf sections in a similar orientation, ensuring that they did not overlap. A coverslip was placed carefully on top, aligned over the sections and, using gentle pressure, the DAPI solution was allowed to spread by capillary action to eliminate bubble formation. Using a light coat of clear nail polish, the edges of the slides were sealed to prevent the coverslip or the sections from moving. Slides were studied under an Olympus BX53 microscope with X-CITE 120 Q fluorescence illumination (Olympus Life Science Solutions, Waltham, MA, USA) connected to a Olympus DP73 microscope digital camera. The midrib areas of several representative tissue sections per plant sample (infected and control leaves) were measured using Olympus cellSens Dimension 1.7 at 100× magnification.

### 2.6 Statistical analysis

Measurements of midrib areas of CBSV-infected and healthy cassava leaf samples were recorded and subjected to one-way analysis of variance (ANOVA) using the GenStat 15th Edition (32 bit) package (Lawes Agricultural Trust, Rothamsted Experimental Station, UK). ANOVA was also used to determine significant differences in mean viral load in the top, middle, and bottom of cassava plants for CBSV-infected and healthy leaf samples.

## 3 Results

### 3.1 CBSD symptom development in source plants

**Fig. 1:**
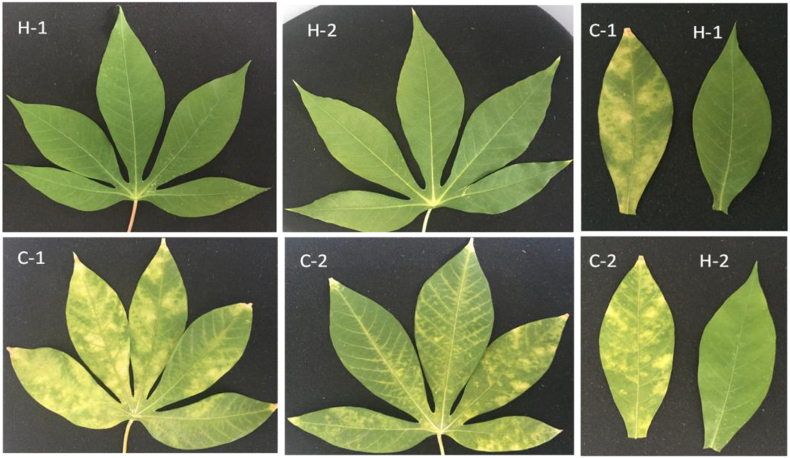
Representative leaf samples of cassava variety Kibandameno used in the immunohistochemical experiments to localize *Cassava brown streak virus* (CBSV). H-1 and H-2 were asymptomatic for CBSV, C-1 and C-2 were CBSV-infected. All infected cuttings showed typical foliar symptoms of cassava brown streak disease, including vein clearing and chlorotic blotches. (For interpretation of the references to color in this figure legend, the reader is referred to the Web version of this article.)

**Fig. 2:**
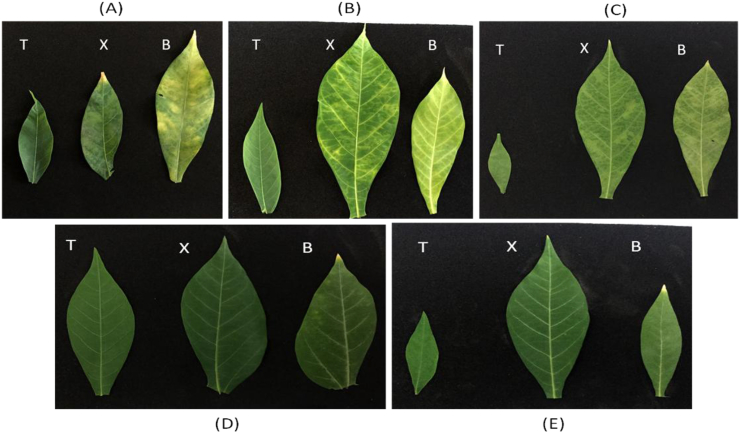
An illustration of three cassava (variety Kibandameno) leaf samples infected with *Cassava brown streak virus* (A–C); and two leaves from disease-free samples (D–E). T, X, and B indicate leaf position; top, middle, and bottom leaves, respectively. Cross-sections of the samples were used for cellular morphology studies. (For interpretation of the references to color in this figure legend, the reader is referred to the Web version of this article.)

All CBSV-infected cassava cuttings established in the screen house developed CBSD symptoms at 5 WAP, similar to those observed under field conditions. Older leaves displayed CBSD foliar symptoms but newly opened leaves did not. Among the major symptoms expressed were chlorotic blotches followed by vein clearing. Chlorosis occurred mainly along and between the smaller veins (Fig. 1). All plants that were asymptomatic in the field were also asymptomatic in the screen house. Sampling to analyze viral localization and plant morphological changes was done on both symptomatic and asymptomatic plants (Fig. 1, Fig. 2).

### 3.2 Screening for CBSV and cassava mosaic begomoviruses

**Fig. 3:**
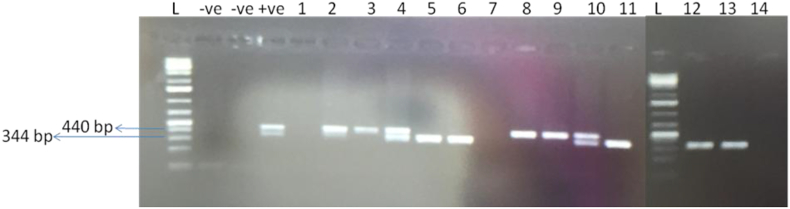
Image of 1% agarose gel showing bands of *Cassava brown streak virus* (CBSV) (344 bp) and *Ugandan cassava brown streak virus* (UCBSV) (440 bp) from leaf samples of cassava variety Kibandameno. The cassava samples were screened using primers developed by Mbanzibwa *et al*. [15]. From left to right: L = GeneRuler™ 1 kb plus ladder, -ve = healthy samples, lanes 1–11 = cassava leaf samples, L = GeneRuler™ 1 kb plus ladder, lanes 12–14 = cassava leaf samples. (For interpretation of the references to color in this figure legend, the reader is referred to the Web version of this article.)

High-quality DNA and RNA were obtained from leaf samples. All samples tested negative for ACMV and EACMV (data not shown). Using the primers CBSDDF2/R, three samples tested positive for the presence of both CBSV and UCBSV, three samples were positive for only UCBSV, and five samples were positive for only CBSV. As expected, the three samples that showed no CBSD symptoms in the field also tested negative for the virus, indicating they were CBSV-free (Fig. 3). The remainder of the experiments used the five cuttings infected with only CBSV or the three cuttings verified to contain no virus (as controls).

### 3.3 CBSV localization

**Fig. 4:**
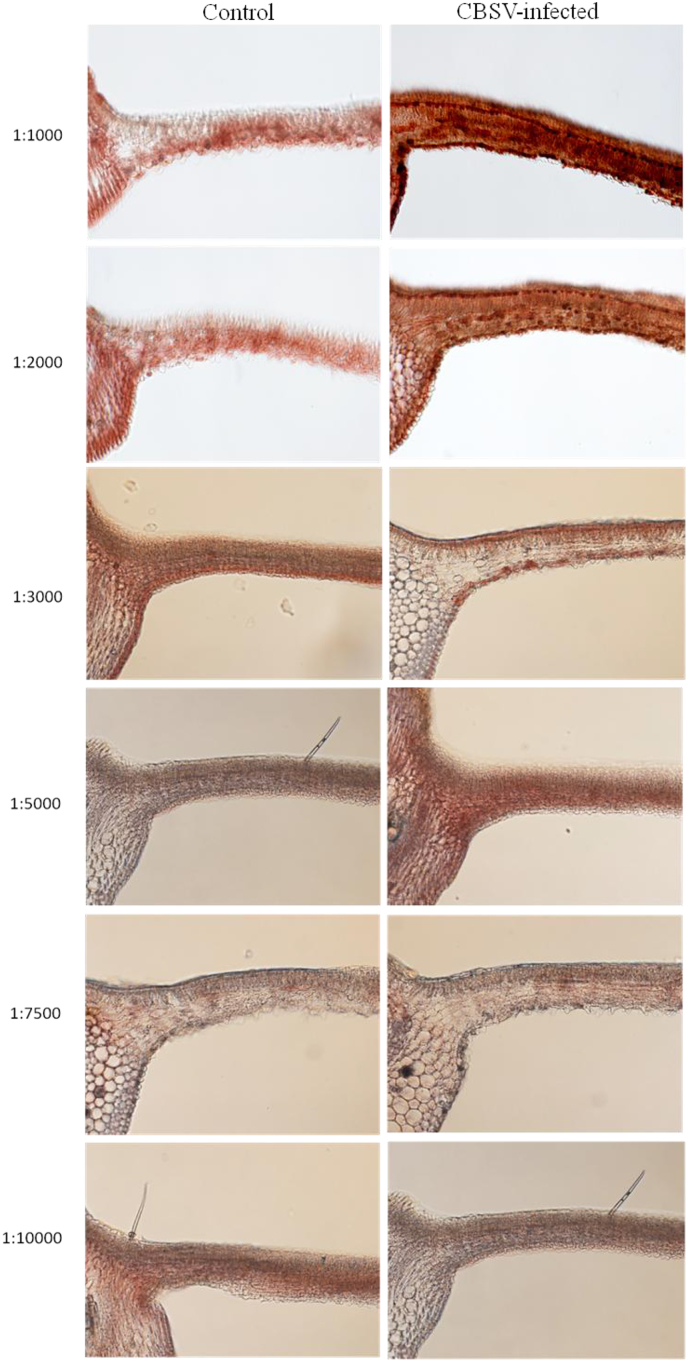
Brightfield images of the cross-sections of cassava (variety Kibandameno) midribs and leaf blades under a fluorescent microscope at 100× magnification. The samples were subjected to immunohistochemical staining using different antibody dilutions (1:1000, 1:2000, 1:3000, 1:5000, 1:7500, and 1:10000) and results compared between samples of the same age infected with *Cassava brown streak virus* (CBSV) and control (uninfected) samples. (For interpretation of the references to color in this figure legend, the reader is referred to the Web version of this article.)

IHC was used successfully as a molecular detection method to localize CBSV within the cells of infected cassava tissues. Staining the tissue sections using different antibody dilutions enabled the determination of the appropriate antibody concentration to use in virus localization. Staining in CBSV-infected cells was intense in the 1:1000 antibody dilution making it difficult to differentiate background with precipitation in infected cells (Fig. 4). However, dilutions 1:3000, 1; 5000, 1:7500 and 1:10000 showed moderate to faint to no staining of infected cells. The procedure worked best using the 1:2000 dilution; we could visualize precipitation in infected cells but not in uninfected cells (AEC system).

**Fig. 5:**
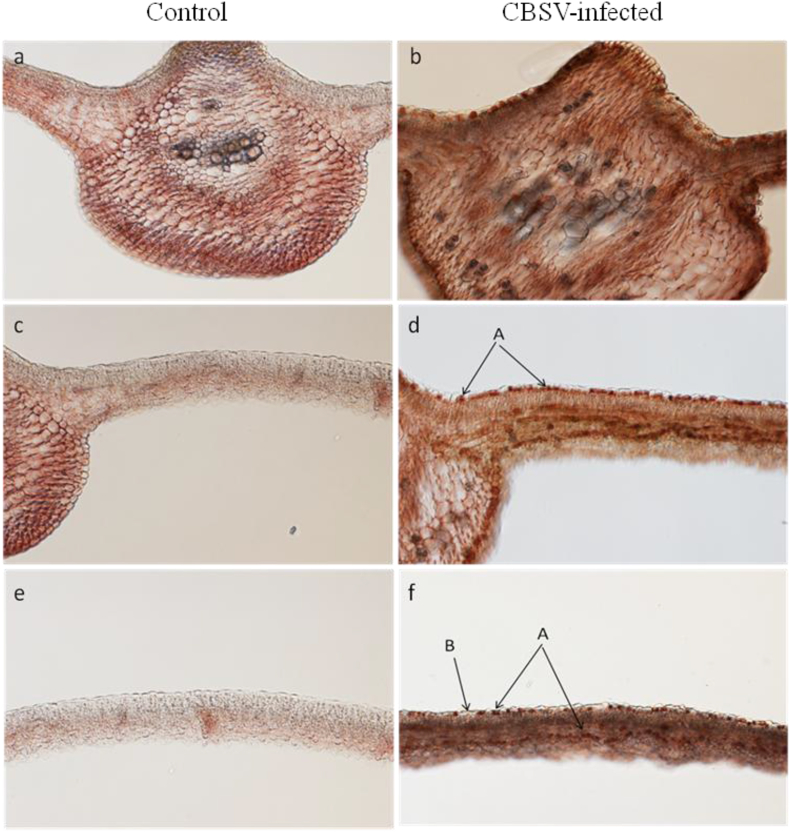
Brightfield images of the cross-sections of cassava (variety Kibandameno) midribs and leaf blades under a fluorescent microscope at 100× magnification. **S**amples were subjected to immunohistochemical staining at an antibody dilution of 1:2000 and results compared between samples of the same age infected with *Cassava brown streak virus* (CBSV) and uninfected (control) samples. Samples infected with CBSV contained cells showing immunohistochemical precipitation (A) and cells that appear virus-free (B). (For interpretation of the references to color in this figure legend, the reader is referred to the Web version of this article.)

**Fig. 6:**
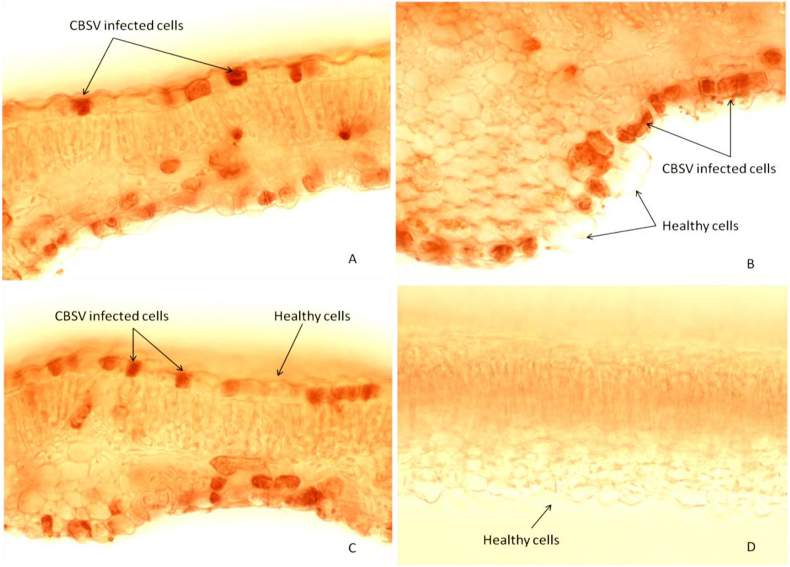
Localization of *Cassava brown streak virus* (CBSV) in infected cassava (variety Kibandameno) tissue samples along the leaf blade as seen under 400× magnification by brightfield microscopy. Locations of CBSV-infected cells are indicated by precipitation in the 3-amino-9-ethyl carbazole (AEC) detection system in infected leaves (A–C), whereas the healthy control leaf (D) shows no precipitation. (For interpretation of the references to color in this figure legend, the reader is referred to the Web version of this article.)

CBSV-infected cells showed staining/precipitation in the phloem and along the adaxial and abaxial epidermal cells all around the leaf blades and central veins, whereas control (uninfected) leaves did not (Fig. 5). Precipitation in infected cells was observed clearly under 400× magnification (Fig. 6). Control samples showed no precipitation in cells due to the absence of CBSV as an antigen (Fig. 6D); however, in some cases, background staining (see control in Fig. 5) occurred when the incubation times of reagents were not optimal or when samples were not appropriately quenched. Peroxidase activity in plants is difficult to quench and some background is expected in these assays even with quenching. Precipitation in cells occurred mostly in the upper and lower epidermis, some in the spongy mesophyll, and rarely in the palisade mesophyll of infected tissue samples (Fig. 6).

**Fig. 7:**
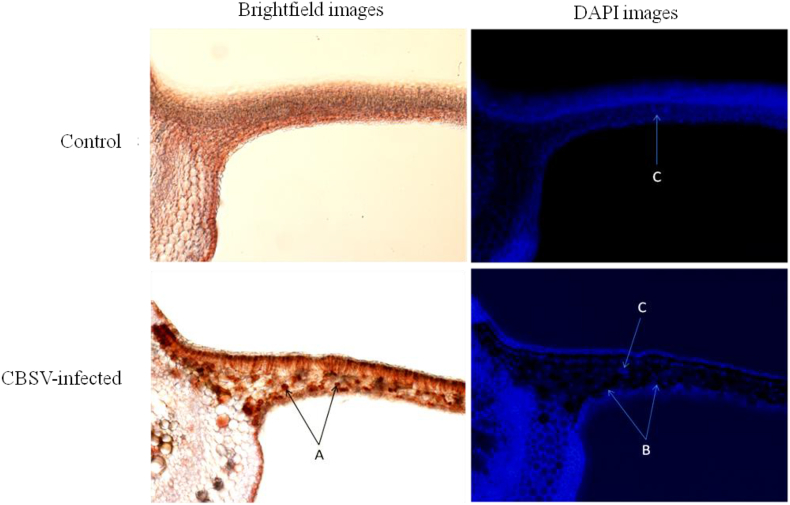
Cross-sections of cassava (variety Kibandameno) midrib and leaf blade under a fluorescent microscope at 100× magnification. Immunohistochemical precipitation is seen in *Cassava brown streak virus*-infected (CBSV-infected) cells (A) and their equivalent DAPI (4′-6′-diamidino-2-phenylindole) images, which show dark cells (B) and fluorescence in the nucleus of uninfected cells (C) in a control (uninfected) plant. (For interpretation of the references to color in this figure legend, the reader is referred to the Web version of this article.)

The staining/precipitation observations were confirmed by comparing the images under brightfield illumination and their equivalent DAPI filter images to identify which cells were infected and which were uninfected (Fig. 7). All CBSV-infected cells showed clear precipitation under fluorescent microscopy.

### 3.4 Cellular morphological changes of CBSV-infected cassava plants

**Fig. 8:**
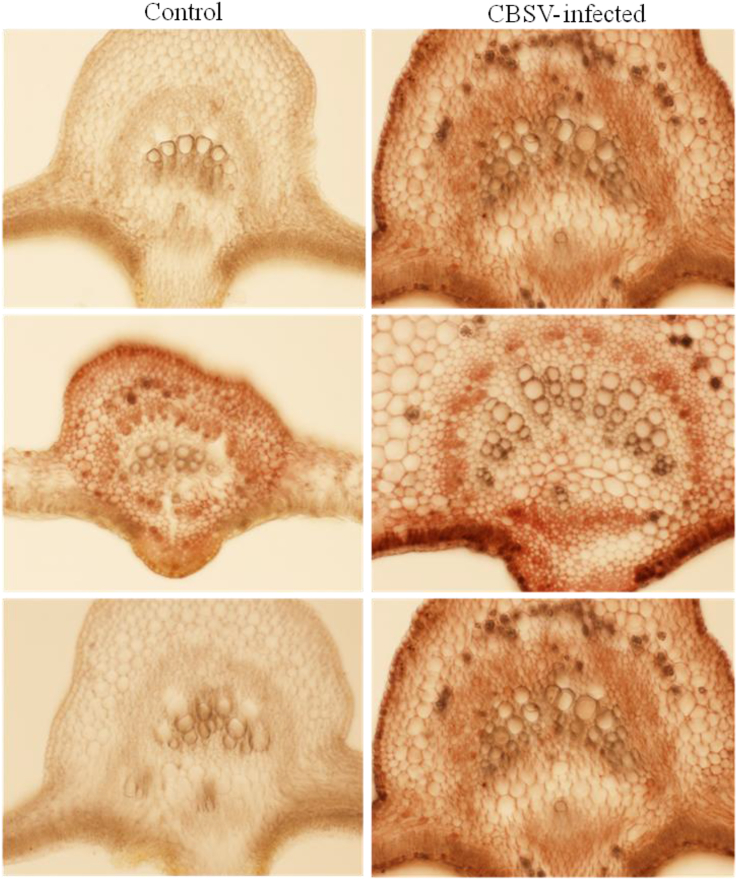
Representative cross-sections of cassava (variety Kibandameno) midribs and leaf blades infected with *Cassava brown streak virus* (CBSV) or uninfected controls. All midrib samples were taken from the middle leaves of cassava plants to illustrate the differences in midrib sizes within the same field of view under 100× magnification. (For interpretation of the references to color in this figure legend, the reader is referred to the Web version of this article.)

Microscopic examination of the cross-sections of cassava leaves revealed differences in the sizes of the midribs between infected and control samples (Fig. 5, Fig. 8), possibly due to differences in thickness of the vascular bundles between infected and control leaves.

**Fig. 9:**
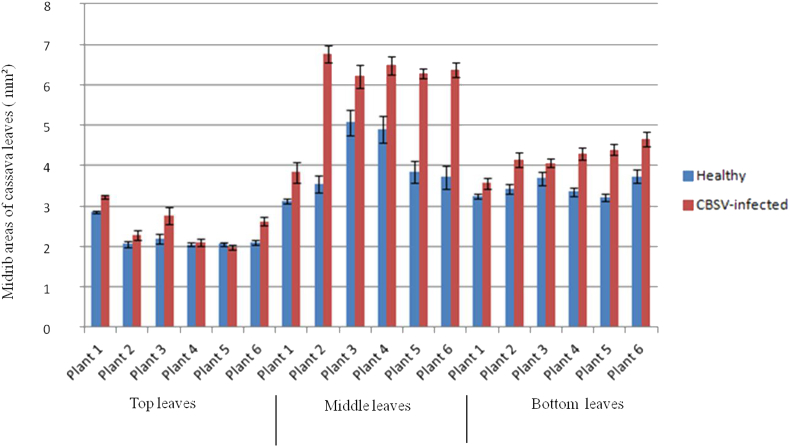
Average midrib areas (mm^2^) calculated using the cross-sectioned leaves of cassava (variety Kibandameno) infected with *Cassava brown streak virus* (CBSV) and uninfected (healthy) controls. Leaves located at the top, middle, and bottom of the cuttings were analyzed separately. The mean area of the midrib from each leaf position was determined using measurements of 10 cross-sections for each sample. A total of six plants were used for cellular morphology studies. Error bars are standard errors of the means (SEM). (For interpretation of the references to color in this figure legend, the reader is referred to the Web version of this article.)

Two main observations were made in this study. First, the localization of CBSV-infected cells was generally higher in the middle and lower leaves compared with the top leaves; second, CBSV-infected cassava leaf samples had significantly larger midrib areas compared with control leaf samples. The mean areas of leaf midribs showed that, whether infected or healthy, top leaves generally had the smallest midribs (cross-section of 2.36 mm^2^) followed by bottom leaves (3.81 mm^2^). The largest midribs were in the middle leaves (5.01 mm^2^). Average midrib areas of healthy leaves ranged from approximately 2.0 to 5.0 mm^2^, whereas samples infected with CBSV had midrib areas that ranged between approximately 2.0 to 6.8 mm^2^ (Table 2). Top-leaf average midrib areas of healthy plants were significantly smaller (2.22 mm^2^) compared with CBSV-infected plants (2.50 mm^2^) (*P* < 0.001). Control and infected samples also differed significantly between middle and bottom leaves (*P* < 0.001) (Fig. 8). In general, for all leaf positions, midrib areas were significantly (*P* < 0.001) greater for CBSV-infected than control leaves (Fig. 9).

**Table 2:** Average area (mm^2^) of the midribs of leaves from three positions (top, middle, and bottom) on cuttings from leaves of plants infected with *Cassava brown streak virus* (CBSV) and controls (uninfected). Mean midrib areas were calculated from 10 cross-sections of each plant and subjected to analysis of variance (ANOVA).

Treatment		Top leaf	Middle leaf	Bottom leaf
Healthy	Plant 1	2.855	3.115	3.244
	Plant 2	2.059	3.530	3.427
	Plant 3	2.193	5.063	3.684
	Plant 4	2.049	4.891	3.350
	Plant 5	2.062	3.840	3.204
	Plant 6	2.103	3.711	3.731
CBSV-infected	Plant 1	3.237	3.833	3.559
	Plant 2	2.286	6.766	4.147
	Plant 3	2.764	6.211	4.063
	Plant 4	2.097	6.475	4.284
	Plant 5	1.976	6.286	4.397
	Plant 6	2.620	6.365	4.662
Mean		2.359	5.007	3.813
LSD		0.162	0.389	0.176
*P*-value		0.001	<0.001	<0.001
% CV^a^		20.9	23.6	14

a CV, coefficient of variation.

All samples used in the study produced similar results, and therefore confirmed that CBSV can be detected using immunohistochemical reaction within the tissues of infected cassava plants, and can be used to explore the virus distribution within infected plants.

## 4 Discussion

In this study, we investigated the cellular localization of CBSV and examined morphological changes in the leaf tissues of infected cuttings of the CBSV-susceptible cassava variety Kibandameno. Our study used immunohistochemical staining and microscopy to investigate differences between leaves at three positions in CBSV-infected and non-infected (control) plants. Immunohistochemical reactions of the samples showed precipitation in CBSV-infected cells but not in CBSV-free tissues, demonstrating successful immunohistochemical localization of CBSV. Microscopic inspection of cross-sectioned leaves showed significantly larger (*P* < 0.001) midribs in CBSV-infected leaves compared with control leaves.

The great advances in plant cell biology have created opportunities for detection methods to locate proteins *in situ* at the cellular level [33]. Immunolocalization and various imaging approaches are increasingly utilized in plant cell biology for a variety of purposes including visualizing molecules of low molecular weights, organelle signaling events, and the interactions of proteins in specific tissues [34]. Potyviruses (including CBSV) move intercellularly through plasmodesmata-associated structures that are coordinated by the interaction of cylindrical inclusion proteins and the third protein–ORF PIPO complex [35]. Information on CBSV localization within plant tissues is currently insufficient to make inferences.

The presence of CBSV in the cells of infected tissues was indicated by precipitation in the cells. We confirmed viral presence with brightfield and corresponding DAPI images. Our prediction was that precipitation occurs where CBSV resides; and that it most likely resides in the cytoplasm of cells due to the nature of this RNA virus. DAPI binds to the adenine–thymine (A–T) regions of nucleic acids, and fluoresces under fluorescent illumination. Precipitation in infected cells restricts DAPI penetration to the nucleus. As a result, images produced under fluorescent illumination show darkness (in non-illuminated nuclei/cells). We found that CBSV was not only localized in the phloem but also in the abaxial and adaxial epidermal cells of the tissues of infected cassava leaves. Our observations confirmed that IHC successfully detected CBSV in the infected tissues of cassava. However, the potential for artefactual staining [36] associated with such detection methods must be considered. Artefacts typically occur because plants have a broad range of strategies to protect themselves from biotic and abiotic stresses; for example, endogenous peroxidase serves as a defense mechanism [37] and is difficult to quench, resulting in some background staining.

Microscopy revealed high precipitation in cells located in the middle and bottom leaves of infected plants and much less or no precipitation in young leaves. This observation concurs with a recent study by Ogwok *et al*. [38] reporting that both CBSV and UCBSV accumulated least in young leaves closer to top of the canopy, and gradually increased down the plant, with the highest viral loads in the oldest leaves farthest from the apex. CBSV may also cause the expansion or increase in the size of leaf midribs. Studying the cellular components of CBSV-infected cassava leaves we found significantly larger (*P* < 0.001) midrib sizes in infected compared with control leaves. Understanding the physiological changes caused by CBSV can help us understand how resistance or mechanisms of control can be directed. Understanding which developmental cues are affected and how morphological differences can be used as indicators of viral resistance or tolerance will help in the design or selection of better genetic material.

Molecular detection of CBSV has not been fully explored and its application is likely to improve our understanding of virus–plant interactions and CBSV pathogenicity. In this study, the molecular detection of CBSV in tissues of infected cassava leaves using IHC was successful. To the best of our knowledge, this is the first report of immunodetection of CBSV from infected cassava tissues.

Plants subjected to external stress usually develop mechanisms in response, such as conserving resources used for growth and reproduction to minimize damage [39]. Globally, agricultural crops are widely affected by abiotic stresses including salinity, cold, heat, and nutrient deprivation, all of which can reduce or even decimate the yields of major crops [40]. Plants must also defend themselves against biotic stresses such as pests and pathogens (e.g. bacteria, fungi, viruses, and nematodes) [41]. These stresses often trigger cellular and molecular response systems to prevent further damage and ensure survival, but can reduce yields or stunt plant growth [42].

Several studies have explored cassava responses to such abiotic stresses as soil salinity [43] and drought [44]. However, plant responses to viral infection, specifically morphological responses at the cellular and tissue levels, remain poorly studied. CBSD produces a variety of obvious foliar symptoms that include mottling, browning, early leaf senescence, mosaic, misshapen and twisted leaflets, and an overall reduction in size of leaves and the plant as a whole [45]. Diseased plants contain less chlorophyll compared with healthy plants due to either the inhibition of chlorophyll synthesis or the destruction of chloroplasts [46], which can in turn result in chlorosis. All these changes in infected cassava plants may affect storage in the roots by influencing nutrient transport (specifically sugar), carbohydrate levels, and the amounts of various sugars in the phloem [47] or storage organs [48]. Olesinski *et al*. [49] reported that viruses can cause significant changes in photosynthetic storage and export, affecting the accumulation of secondary metabolites such as cyanide that in turn allows the induction of resistance mechanisms by the infected plant—an important factor in plant defense. There is abundant evidence that cassava plants respond to infection with structural and physiological modifications [50], including changes in the chloroplasts, altered carbon metabolism, and the accumulation of starch grains, which result in chlorosis and necrosis [51]. Transcriptome profiling has revealed the alteration of genes responsible for antiviral defense responses [52,53] and may be one of the reasons for these cellular morphological changes.

The increase in diameter and areas of midribs in general may be caused by CBSV infection in cassava. The midribs comprise vascular bundles (xylem and phloem) that are responsible for transporting water and nutrients from root to shoot and leaf to root [54]. Reduced sugar synthesis and nutrient levels in leaves of CBSV-infected plants may stimulate increases in the size of vascular systems as a means to maximize water and nutrient uptake as well as storage. Our observations of the morphological differences in CBSV-infected midribs may be just one of many modifications that result from infection. Changes to cassava metabolism caused by a pathogen may result in many more visible phenotypic and biochemical differences.

## 5 Conclusion

IHC or immunolocalization is generally an important and efficient tool with which to understand genome organization, molecular structures and their respective functions, and protein-associated antigens and pathogens. Here, CBSV was successfully localized with the IHC detection method using 1:2000 dilution of rabbit-*anti*-CBSV CP antibody. We suggest the use of IHC in conjunction with similar autoradiography experiments for improving studies of virus localization. Cross-sections of CBSV-infected leaves revealed significant morphological differences compared with healthy plants. Further research is needed to understand why these changes occur and what implications they have for the affected plant. This in turn will give insight on the processes involved in CBSV infection and establishment of the virus within the plant, contributing to strategies with which to manage CBSD.

## Declarations of competing interest

None.
